# Serum BDNF, VEGF, and EGF Levels in Pregnant Women with Anxiety and Depression

**DOI:** 10.3390/ijms27198885

**Published:** 2026-10-06

**Authors:** Philippe Leff-Gelman, Ismael Mancilla-Herrera, Amado Pérez-Molina, Mónica Flores-Ramos, Blanca Farfan-Labonne, Fausto Manuel Cruz-Coronel, María del Pilar Meza-Rodríguez, Ignacio Camacho-Arroyo, Carlos S. Cruz-Fuentes

**Affiliations:** 1 Instituto Nacional de Perinatología, Mexico City 11000, Mexico; pleff49@ymail.com (P.L.-G.); mezapilar@yahoo.com (M.d.P.M.-R.); 2 Instituto Nacional de Psiquiatría, Ramón de la Fuente Muñiz, Av. Xochimilco #101. Col. San Lorenzo, Huipulco, Tlalpan, Mexico City 14370, Mexicoflores_ramos@hotmail.com (M.F.-R.); 3 Hospital General de México, Eduardo Liceaga, Mexico City 06720, Mexico; 4 Unidad de Investigación en Reproducción Humana, Instituto Nacional de Perinatología-Facultad de Química, Universidad Nacional Autónoma de México, Mexico City 04510, Mexico

**Keywords:** anxiety, depression, pregnancy, brain-derived neurotrophic factor (BDNF), vascular endothelial growth factor (VEGF), epidermal growth factor (EGF)

## Abstract

Antenatal anxiety and depression frequently co-occur, negatively affecting maternal and offspring outcomes, yet their biological correlates remain insufficiently characterized. This cross-sectional study examined associations between anxiety and depressive symptoms and serum concentrations of brain-derived neurotrophic factor (BDNF), vascular endothelial growth factor (VEGF), and epidermal growth factor (EGF), proteins implicated in pregnancy-related vascular adaptations whose dysregulation has been associated with placental dysfunction and negative perinatal outcomes. A total of 148 women with uncomplicated second- and third-trimester pregnancies were assessed using the Hamilton Anxiety Rating Scale and Hamilton Depression Rating Scale. Serum BDNF levels were quantified by enzyme-linked immunosorbent assay, whereas VEGF and EGF were measured using a multiplex bead-based immunoassay during late pregnancy. Based on scale scores, participants were classified as controls, women with anxiety without depressive symptoms, or women with anxiety and depressive symptoms. In the between-group comparisons, women with clinically significant anxiety exhibited higher BDNF and EGF levels than controls. Regression analyses indicated that anxiety severity was positively associated with BDNF, VEGF, and EGF levels (all *p* < 0.05), whereas depressive symptom severity was negatively associated with BDNF levels (*p* < 0.05). These findings suggest distinct associations of antenatal anxiety and depressive symptoms with circulating growth factors, particularly BDNF, and warrant further investigation of vascular–immune–neurotrophic pathways potentially involved in perinatal mental health.

## 1. Introduction

Anxiety, along with depression, represents one of the most prevalent and clinically significant psychiatric conditions experienced during the perinatal period [1,2,3]. Prevalence estimates indicate that around 20% to 29% of women experience at least one anxiety disorder during pregnancy or the postpartum period [4,5].

Although anxiety and depressive symptoms can be common and transient during pregnancy [6], if they persist and are untreated, they may become clinically significant, negatively affecting quality of life and impairing the daily functioning of the pregnant woman [7,8]. More importantly, both disorders not only negatively impinge upon maternal well-being but also extend beyond delivery, leading to adverse obstetric, neonatal, and developmental outcomes, including poorer social-emotional, cognitive, language, and adaptive functioning in offspring [9,10,11,12]. Despite growing recognition of the detrimental role of depressive and particularly anxiety symptoms during the perinatal period, the psychobiological mechanisms underlying their manifestation remain incompletely understood.

Current evidence defines pregnancy as a complex, multifactorial model of significant physiological changes, in which neuroendocrine, immune-inflammatory, placental-related processes [13], and sociopsychological factors [14] interact and extend into the postpartum period. During pregnancy, extensive remodeling occurs, marked by dynamic adaptations in immune tolerance, hematopoiesis, angiogenesis, endothelial function, and placental vascular development [15,16]. Collectively, these processes establish the physiological context in which perinatal depression and anxiety may manifest.

Most biomedical research on perinatal mental health has investigated classical inflammatory cytokines as potential biomarkers of related diseases or disorders [17,18,19]. Regarding depressive symptoms, a recent systematic review highlighted the role of inflammatory dysregulation, with the most recurrent altered signals across pregnancy and the postpartum period being IL-6, IL-8, CRP, and TNF-α [20]. However, for perinatal anxiety, other distinctive immune profiles have been detected, suggesting that anxiety-related biology may not be fully reducible to depressive symptomatology [21].

In any case, the roles of many other circulating molecules traditionally classified as growth factors, angiogenic factors, or those related to endothelial function have received comparatively less attention. Notably, some of these proteins—primarily recognized as factors involved in proliferation, differentiation, and tissue repair—constitute a plausible biological interface linking perinatal affective/anxiety symptoms with placental, vascular, and neurodevelopmental processes. On the other hand, some findings suggest that disruptions in growth-factor signaling indicate both nonspecific inflammatory activity and impaired vascular and neurotrophic adaptation during pregnancy and the postpartum period.

In particular, brain-derived neurotrophic factor (BDNF) has been proposed as a biologically significant molecule during pregnancy, positioned at the intersection of placental function, fetal development, maternal metabolism, and stress-related neuroendocrine regulation [22,23]. BDNF protein and its receptor TrkB are expressed in the placenta and fetal tissues, where they participate in processes like stimulation and survival of trophoblasts and the implantation of the blastocyst, essential events in subsequent placental development and fetal growth [24]. Moreover, alterations in the BDNF/TrkB system have been described in pregnancy-related complications such as preeclampsia, gestational diabetes, intrauterine growth restriction, and preterm delivery [25]. Collectively, these findings indicate that, beyond its established role in the development and function of the nervous system, BDNF can also be a mediator of fetoplacental development and pregnancy adaptation [26,27]. Beyond its role as a neurotrophic factor, the protein has also been implicated in mood regulation during pregnancy. Therefore, reduced BDNF levels have often been linked to maternal perinatal depression, either in early pregnancy following antepartum depression [28], post-traumatic stress disorder with comorbid depression [29], or in postpartum depression [30,31,32,33]. However, much less is known about its relation to anxiety; nonetheless, anxious and depressive symptoms are frequently comorbid [4,6].

Vascular endothelial growth factor A (VEGF-A) is another molecular candidate to explore due to its established roles in endothelial cell survival, regulation of the blood–brain barrier, and vascular development [34,35]. The expression of vascular endothelial growth factor (VEGF) and its receptors VEGFR-1 (Flt-1) and VEGFR-2 (KDR) plays critical roles in promoting normal placental angiogenesis [36]. A longitudinal study indicated that serum VEGF-A concentrations decrease during the peripartum, a decline particularly pronounced in women experiencing antenatal and postpartum depression [17]. However, the relationship between VEGF-A and perinatal anxiety remains insufficiently characterized.

Epidermal growth factor (EGF) may also be particularly relevant during the perinatal period, when significant placental and vascular adaptations occur. Accumulating evidence suggests that human trophoblast survival and invasive capacity are linked to intercellular signaling by peptides related to epidermal growth factor (EGF). Peptide members of the EGF signaling system induce downstream signaling by binding to receptor tyrosine kinases of the human EGF receptor (EGFR)/ERBB family [37]. The placenta has the highest expression levels of the EGF receptor protein in the human body [38]. However, the role of the EGF signaling system remains comparatively unexplored in relation to perinatal depression and anxiety [39].

The measurement of serum and plasma proteins provides a minimally invasive approach to investigating biological processes associated with psychiatric conditions and may serve as proxies that complement clinical assessment. Alterations in peripheral levels of neurotrophic factors, such as BDNF, VEGF, or EGF, can help characterize disruptions in pathways related to neuronal plasticity, vascular regulation, and vascular–immune–neurotrophic pathways.

Accordingly, the present study aimed to examine serum levels of these distinct peripheral growth factors in relation to depression and anxiety symptoms during pregnancy. Particularly, peripheral alterations in the concentrations of BDNF have been extensively investigated as a potential biomarker for disease state, severity, or treatment response in relation to clinical depression. However, although perinatal depression has been frequently associated with reduced serum levels of brain-derived neurotrophic factor [40], the relationship between the neurotrophin and maternal anxiety remains significantly less characterized, with just a single report showing elevated maternal serum BDNF concentrations associated with higher scores on the Spielberger State-Trait Anxiety Inventory (STAI) [41]. Grounded in these contrasting neurotrophic patterns, we hypothesized that circulating BDNF levels would manifest opposing trajectories when evaluating maternal depressive versus anxiety symptomatology in our cohort. Furthermore, to elucidate the broader growth factor network underlying perinatal distress, we established secondary hypotheses evaluating whether maternal anxiety and depression are distinctively associated with circulating levels of vascular endothelial growth factor and epidermal growth factor.

By analyzing these biologically interconnected systems, this work aims to advance the understanding of the vascular–immune–neurotrophic pathways underlying affective vulnerability during pregnancy and the postpartum period.

## 2. Results

### 2.1. Sociodemographic Characteristics of the Recruited Pregnant Population

At the time of assessments (third trimester of their pregnancy), participants had a mean age of 25.2 ± 6.0 years and a mean gestational age of 34.8 ± 4.0 weeks. Mean body weight was 67.2 ± 10 kg, with a mean body mass index (BMI) of 27.8 ± 3.7 kg/m^2^. Most participants were either cohabiting (39.2%) or divorced (26.4%), had completed either middle (33.1%) or high school (32.4%), and were primarily engaged in home labor (43%).

### 2.2. Clinical Groups

Since anxiety and depressive symptoms were the focus of the present analyses, participants were categorized according to the Hamilton Anxiety (HARS) and Depression (HDRS) Rating Scale scores. Pregnant women with both HARS and HDRS scores in the non-clinical range (≤7) were defined as the control group (CTRL; *N* = 35) and used as the reference category for further analyses. In contrast, those meeting the threshold for at least mild anxiety symptoms (HARS ≥ 8) were classified into a global anxiety group (ANX; *N* = 113; HARS range: 16–39). The ANX group was subsequently subdivided according to depressive symptom burden, yielding an anxiety-only subgroup with HDRS scores ≤ 7 (ANX − DEP; *n* = 66; HDRS range: 6–7) and an anxiety and depressive high-symptom subgroup (ANX + DEP; *n* = 47; HDRS range: 20–51) (Table 1). HARS and HDRS were moderately and positively correlated (Spearman’s ρ = 0.555, *p* < 0.001), revealing the frequent parallel manifestation of anxiety and depressive symptoms.

A one-way ANOVA showed no significant between-group differences in body weight or BMI. However, age differed significantly across groups (*F*(2, 145) = 6.86, *p* = 0.001, η^2^ = 0.07), with women belonging to CTRL group exhibiting a higher mean age than either ANX − DEP and ANX + DEP groups (Tukey post hoc for ANX − DEP group: *b* = −4.41, SE = 1.20, *t* = −3.69, *p* < 0.001; Tukey post hoc for ANX + DEP group: *b* = −3.24, SE = 1.28, *t* = −2.54, *p* = 0.012).

Although the mean gestational age at assessment between groups of comparison did not reach statistical significance (*F*(2, 145) = 2.81, *p* = 0.064, η^2^ = 0.07), the distribution of evaluations across gestational-week categories was not uniform among groups: χ^2^ (8, *N* = 148) = 26.21, *p* = 0.001, Cramer’s *V* = 0.30. Specifically, the ANX − DEP group was represented by mothers evaluated at later gestational weeks compared to the CTRL and ANX + DEP groups.

Regarding sociodemographic characteristics (Table 2), no significant between-group differences were observed in marital status (χ^2^(6) = 3.64, *p* = 0.725), educational levels (χ^2^(10) = 4.25, *p* = 0.935), or employment status (χ^2^(10) = 11.64, *p* = 0.310).

### 2.3. Serum Growth Factor Levels Across Gestational Weeks During the Third Trimester of Pregnancy

We examined the potential influence of gestational age on the observed circulating BDNF, EGF, and VEGF concentrations. As shown in Figure 1A–C and backed by the regression analyses, serum levels of the three growth factors did not differ substantially across the trimester examined, either overall or between groups. The only exception was EGF in the ANX − DEP group, where the linear regression slope was statistically significant (R-squared = 6.13 × 10^−2^, F(1,64) = 4.18; *p* = 0.045).

### 2.4. Serum Levels of BDNF, VEGF and EGF in the Third Trimester of Gestation as a Function of Anxiety

Pregnant women with anxious symptoms of clinical relevance (ANX), irrespective of the presence of depressive symptoms (*n* = 113), showed higher circulating levels of BDNF, EGF, and VEGF compared with the control group (*n* = 35) (Figure 2). BDNF: ANX 18.2 ± 5.7 vs. CTRL 13.6 ± 4.3 ng/ml; Welch’s *t*(81.9) = −4.55, *p* < 0.001. EGF: ANX 91.3 ± 27.2 vs. CTRL 79.4 ± 21.8 pg/ml; Welch’s *t*(69.8) = −2.66, *p* = 0.010. VEGF: ANX 152.1 ± 24.7 vs. CTRL 142.8 ± 23.3 pg/ml; Welch’s *t*(59.6) = −1.98, *p* = 0.052.

In the case of BDNF, peripheral levels differed significantly across the three categories evaluated, with a significant main effect of the diagnostic group: *One-factor ANOVA* (*F*(2, 119) = 13.9, *p* < 0.001). The effect was maintained after controlling for age: ANCOVA F(3, 118) = 9.7, *p* < 0.001, partial η^2^ = 0.158.

Post hoc comparisons, using Tukey’s HSD test, indicated that BDNF levels were significantly higher in the ANX − DEP group with respect to the CTRL group (mean difference = −5.7, SE = 1.2, *t*(118) = −4.7, *p* < 0.001, Cohen’s *d* = 1.10, 95% CI [0.61, 1.59]). No significant differences were observed between the CTRL and ANX + DEP groups (mean difference = −2.6, SE = 1.2, *t*(118) = −2.11, *p* = 0.092). BDNF levels were also significantly higher in the ANX − DEP group than in the ANX + DEP group (mean difference = 3.1, SE = 1.1, *t*(118) = 2.8, *p* = 0.016, Cohen’s *d* = 0.60, 95% CI [0.17, 1.04]).

Similarly, in the case of EGF, serum levels of this factor were unequal among the groups: *One-factor ANOVA* (*F*(2, 145) = 3.6, *p* = 0.03). The main effect of the diagnostic group was maintained after controlling for age: ANCOVA *F*(3, 144) = 3.14, *p* = 0.027, partial η^2^ = 0.031. Tukey-adjusted post hoc comparisons showed a significant mean difference between the ANX − DEP and CTRL groups (mean difference = −14.5 ± 5.4, *t*(145) = −2.66, *p* = 0.024) but not for the ANX + DEP group (mean difference = −8.4 ± 5.8, *t*(145) = −1.44, *p* = 0.32).

Similarly, no statistically significant differences were found in serum VEGF levels among the study groups under a two-tailed hypothesis. *One-factor ANCOVA*: *F*(3, 144) = 1.9; *p* = 0.14.

### 2.5. Linear Regression Analyses

General linear models with robust standard errors and bias-corrected and accelerated bootstrap confidence intervals based on 10,000 resamples were used to examine associations between anxiety symptom severity and serum BDNF, EGF, and VEGF concentrations. In the unadjusted analyses, HARS scores were positively associated with all three proteins: BDNF (*B* = 0.163, β = 0.320, *p* < 0.001), EGF (*B* = 0.509, β = 0.217, *p* = 0.009), and VEGF (*B* = 0.412, β = 0.191, *p* = 0.017). The models explained 10.2%, 4.8%, and 3.6% of the variance in BDNF, EGF, and VEGF concentrations, respectively, with adjusted *R*^2^ values of 0.095, 0.041, and 0.030.

Covariate adjustment increased the variance explained by each model. For BDNF, including HDRS scores and maternal age increased *R*^2^ to 0.174 (adjusted *R*^2^ = 0.153), and the overall model remained significant, *F*(3, 118) = 8.27, *p* < 0.001. Within this model, depressive symptom severity was negatively associated with BDNF concentrations (*B* = −0.132, β = −0.281, *p* = 0.006). For EGF, gestational week was included because of its association with EGF concentrations in the ANX − DEP subgroup. After adjustment, HARS scores remained positively associated with EGF (*B* = 0.413, β = 0.178, *p* = 0.036), with the model explaining 6.5% of the variance (adjusted *R*^2^ = 0.052). Similarly, the positive association between HARS scores and VEGF persisted after adjustment for maternal age (*B* = 0.364, β = 0.168, *p* = 0.043); this model explained 4.3% of the variance (adjusted *R*^2^ = 0.030).

Because the primary analysis assessed the regression between HARS anxiety scores and serum BDNF concentrations, adjusting for HDRS depressive symptoms and covariates such as maternal age, we conducted an approximate sensitivity analysis under standard linear regression assumptions to determine the minimum detectable effect size at a specified power level. We tested HARS as the sole predictor, and the full model included HARS, HDRS, and maternal age. We set the significance level at α = 0.05, with 80% power and a total sample size of 122. The incremental contribution of HARS scores to serum BDNF concentrations, after adjustment for HDRS scores and maternal age, yielded an effect size of Cohen’s f^2^ = 0.065, corresponding to a partial correlation of approximately 0.25. Increasing the power to 90% resulted in a minimum detectable effect size of f^2^ = 0.088, corresponding to a partial correlation of approximately 0.28.

## 3. Discussion

The present work investigated the relationship between the expression of depressive and anxiety symptoms in expectant mothers and circulating antenatal levels of BDNF, VEGF, and EGF, three protein factors involved in critical physiological events underlying normal vascular adaptations during pregnancy, whose dysregulation has been associated with placental dysfunctions and negative perinatal outcomes (e.g., BDNF: [22]; VEGF: [36]; EGF: [37,42]). Particularly, BDNF exerts diverse physiological effects that support an overlapping role in both reproductive biology and mood regulation [43].

Anxiety of clinical relevance during pregnancy has been linked to several adverse postpartum outcomes, like increased odds of preterm birth, low birth weight, or a reduction in breastfeeding [10,44]. Moreover, it is associated with the behavioral expression of negative attitudes during motherhood, altering the maternal-fetal quality of attachment [11,45]. In this sense, a feature of concern, worth mentioning, was the high prevalence of clinically significant anxiety and depressive symptoms detected in our sample, which may be associated with the sociodemographic characteristics of the population studied. Participants in our study were recruited from a major public hospital in Mexico City that primarily serves a segment of the population lacking social security coverage and in which low educational attainment and underemployment are common. This interpretation is consistent with evidence from other Latin American settings. For instance, pregnant women from rural areas of Nicaragua who attended public hospitals or community health centers exhibited a substantially higher prevalence of antenatal anxiety (41%) and depressive symptoms (57%) than pregnant women receiving healthcare in the Netherlands (15% and 6%, respectively), a high-income country [46]. Similarly, an elevated prevalence of both state (59.5%) and trait (45.3%) anxiety was reported among women attending a gynaeco-obstetric clinic in Brazil [47]. Altogether, these findings suggest that socioeconomic disadvantages and disparities in access to healthcare may contribute to the elevated burden of antenatal anxiety observed in certain populations. Other factors, including a history of psychological or psychiatric problems, lower educational attainment, unemployment, and marital difficulties, may further increase this vulnerability [14].

A body of research has examined its relation to psychiatric conditions during the perinatal stage. However, most studies have focused on depression, while considerably less attention has been given to its association with maternal anxiety, despite the noteworthy co-occurrence of depressive and anxiety symptoms during pregnancy [12].

In the case of BDNF, the literature reporting its serum/plasma levels in the perinatal period is extensive and heterogeneous, mostly referring to associated obstetric conditions like preeclampsia, gestational diabetes, hyperemesis gravidarum, or premature delivery [48,49,50].

Circulating BDNF during pregnancy has been reported to be lower than in non-pregnant women [51,52,53]. Nonetheless, data regarding its concentration throughout pregnancy are imprecise. For instance, a sustained decline of the neurotrophin from the first through third trimesters was reported, with a subsequent increase at postpartum [53]; another study detected a slight increase in the protein just during middle and late pregnancy [54]; while another report identified that its levels were generally stable over the period analyzed, with a sustained decline only in relation to preeclampsia [48]. In any case, we did not identify substantial changes in circulating levels during the third trimester examined.

When the cohort of pregnant women was categorized according to severity of anxiety and depressive symptoms, it was evident that the circulating levels of BDNF were significantly elevated in those experiencing anxious symptomatology, with differences especially noticeable in those women without concomitant depressive symptoms. Linear regression analysis supported this finding, showing that HARS scores positively predicted serum BDNF concentrations, accounting for 12.6% of the residual variance. Taken together, these experimental data confirmed our primary hypothesis that greater antenatal anxiety symptom severity is associated with higher circulating BDNF levels.

Nonetheless, the physiological significance of the relationship between anxiety symptom severity and serum BDNF levels is challenging to interpret given the limited availability of comparable data. A systematic review and meta-analysis of all studies published by 2013 in relation to circulating BDNF levels and anxiety disorders found that protein levels were generally decreased among affected individuals [55]. However, the reduction was not consistently seen across the various anxiety disorders examined, being largely attributable to the significantly lowered levels found in OCD patients. In addition, BDNF levels in cord blood samples from newborns of women diagnosed with generalized anxiety disorder were reported to be lower compared with samples from infants of healthy women [56]; however, this finding was not replicated by other researchers [57]. In the study most closely related to the present research [41], Dingsdale et al. initially found no overall correlation between maternal BDNF levels and State-Trait Anxiety Inventory scores in their cohort. Still, when stratified by fetal sex, they observed a significant positive association among mothers of male infants. Therefore, the anxiety symptoms seem to represent a factor of moderate effect size relative to the other predictors.

As cited above, most of the research addressing the relationship between BDNF and psychiatric/psychological ailments in the antenatal stage has centered on clinical depression or exacerbated depressive symptomatology. For example, Fung et al. reported that women with serum BDNF levels in the lowest three quartiles during early pregnancy had a 1.6-fold increased risk of antepartum depression compared to those in the highest quartile [28]. Christian et al. identified an inverse relationship between serum BDNF and depressive symptoms in the third trimester [54]. Similarly, Zhang et al. found in Chinese women that their serum BDNF levels throughout pregnancy were negatively correlated with PHQ-9 scores and a reduced risk of prenatal depressive symptoms [58]. A recent meta-analysis concluded that experimental data support reduced BDNF protein levels in both antepartum and postpartum depression [40]. However, other studies have not observed this negative association [33,51,59].

On the other hand, our finding of a statistically significant reduction in BDNF levels in the subgroup of women with anxiety who also expressed depressive symptoms compared with those with no manifesting affective symptoms of clinical relevance (although not statistically different with respect to controls) can be interpreted in the context of hierarchical linear regression analysis, in which the inclusion of HDRS scores in the model changed the regression coefficients, disclosing instead a negative relation for the depressive symptoms, as hypothesized, while maintaining the positive association for anxiety symptoms. Overall, these results suggest that both symptom types exert a mixed influence on neurotrophin levels in the last trimester of pregnancy.

As stated, we also identified slightly higher levels of both EGF and VEGF in the group of women showing anxiety symptoms but not showing depressive symptoms. At the same time, HARS scores were positive predictors of their serum concentrations.

Both proteins play critical roles in promoting normal placental angiogenesis and nutrient transport, processes especially relevant during the perinatal period, when the placenta and vasculature undergo significant adaptations. Imbalances in VEGF and EGF levels and their receptors have been associated with placental dysfunction and clinical conditions such as gestational diabetes mellitus and eclampsia [36,37,60,61,62]. In particular, elevated VEGF has been linked to increased placental vascularization, which may enhance nutrient availability to the fetus and promote overgrowth. In this context, Mirabelli et al. reported that—as with well-established altered metabolic conditions such as maternal hyperglycemia—serum VEGF and EGF levels were also positive predictors of accelerated fetal growth [63].

Limited data regarding EGF and VEGF in the context of anxiety disorders are available. Roknuzzaman et al. reported elevated serum EGF levels in generalized anxiety disorder (GAD) patients compared to healthy subjects, an increase that was also positively associated with GAD severity, as measured by the GAD-7 [64]. However, to our knowledge, the relationship of these growth factors and perinatal depression or anxiety had not been examined.

Nonetheless, it is worth mentioning some studies that investigated their association in the context of psychosocial stress-related illnesses, which are often linked to anxiety. For example, increased baseline plasma EGF or VEGF levels were identified in Swedish women exhibiting either significant signs of occupational stress or during the period of an extended sick leave as a consequence of having experienced an affective or stress-related mental disorder following chronic stress exposure [65]. Similarly, women with stress-related exhaustion disorder exhibited significantly elevated plasma VEGF and EGF levels compared to healthy women, a pattern that persisted although attenuated after 24 months of follow-up [66]. In contrast, markedly low concentrations of EGF, VEGF, and BDNF were detected in subjects who experienced stress-related exhaustion disorder compared to controls. However, counterintuitively, it was found that anxiety symptoms (as measured by the Hospital Anxiety and Depression Scale) moderately correlated with the three molecules, whereas no correlation was seen with symptoms of depression or burnout [67].

Several limitations of the present analysis may affect the interpretation of the results. First, as noted, the sample included Mexican women with particular socioeconomic features that are not representative of the broader population of pregnant women. Second, the severity of anxiety symptoms was assessed at a single point in time during a specific stage of pregnancy. Moreover, we used a clinical scale with questions targeting general worries, fears, and somatic concerns; however, pregnancy-specific concerns like fear of childbirth or worries about bearing a handicapped child are not included [12,14,68]. Moreover, effect sizes and variance explained by the regression analyses indicate that the observed variation in the levels of the analyzed molecules in relation to anxiety symptoms is modest at most for BDNF and even smaller for the other growth factors. Consequently, a substantial portion of the variance likely arises from unmeasured factors or variables not included in the analysis. Future research with longitudinal designs, multiple and more specific assessments, and verification procedures is warranted.

## 4. Materials and Methods

### 4.1. Design of the Study

The present cross-sectional analytical study was conducted in the Department of Gynecology and Obstetrics at the General Hospital of Mexico (HGM, Dr. Eduardo Liceaga, Mexico City, Mexico) from 2015 to 2017. The study was reviewed and approved by the hospital research and bioethics committees in 2014 (project ID: D1/14/112/04/072, 2014–2017).

Participants were women aged 18–35 years with uncomplicated second- and third-trimester pregnancies, who met the inclusion criteria and, after being informed of the purpose of the study and the required clinical and laboratory procedures, voluntarily agreed to participate. Most participants resided in Mexico City or the surrounding states.

After hospital admission, researchers examined women to collect physical, clinical, and obstetric information. Data gathered included sociodemographic (marital status, education level, and working status), anthropometric (height, weight, and body mass index [BMI]), obstetric variables (maternal age, parity, and mode of delivery), and pregnancy status.

Those individuals consuming psychotropic medication or illicit substances of abuse, with psychiatric disorders (other than major depression and anxiety diagnosed before or during enrollment of patients), obstetric pathologies, acute or chronic infections, and other medical conditions (i.e., neurological, metabolic, cardiovascular, degenerative, neuroendocrine, immune-related disorders), including assisted conception, were excluded from participation in the study at entry.

A total of 148 women in the third trimester (28–40 weeks of gestation) of their pregnancy were included. Participants reported no alcohol consumption, smoking, drug use, or abuse. They were not medicated during the initial medical interviews and blood sampling.

Upon entry, participants underwent second- and third-trimester blood and urine tests, a complete lipid profile, a steroid hormone profile, a thyroid study, and fetal ultrasound. Laboratory tests were approved by the bioethics committee (Bioethics Approval document No. CI/223/14, 2014) and performed at the hospital’s Central Laboratory.

Elimination criteria included the following: patients with incomplete questionnaires; absent or incomplete laboratory tests; inconsistencies in the psychiatric evaluation of mood disorders; missing regular appointments; or abandonment of the protocol.

Women without a history of acute or chronic obstetric complications, acute or chronic illnesses, or neuropsychiatric pathology (other than mood- or anxiety-related complaints) within the past six months were referred to the Psychiatry Department for psychological counseling and psychiatric evaluation.

No associated mental pathologies (i.e., bipolar disorder, schizophrenia, psychosis, and neuropsychiatric disorder, including uncontrolled compulsive eating and obsessive disorders) were identified based on the diagnostic criteria defined in the Diagnostic and Statistical Manual of Mental Disorders, DSM-IV-TR [69]. Mood or affective symptoms were assessed by clinical psychologists applying the Spanish-validated versions of the hetero-reported Hamilton Depression Rating Scale (HDRS) [70] and the Hamilton Anxiety Rating Scale (HARS) [71].

Upon completing the clinician-rated scales, all participants (fasting for 8–12 h) were referred to the clinical laboratory for blood sampling and quantification of serum analytes.

### 4.2. Blood Sampling

Blood sampling was carried out between 7:00 and 9:00 am., under aseptic conditions. Six mL of venous blood was collected in sterile 13 × 100/Vacutainer BD Hemogard Tubes containing Clot Activator/Polymer Gel for serum separation (Becton & Dickinson 367977, Franklin Lakes, NJ, USA). Samples were cooled on ice and allowed to clot for 1 h before serum separation. The serum fraction was obtained by centrifugation at 1600× *g* for 15 min, aliquoted, and stored at −80 °C until testing.

VEGF AND EGF were determined using the immunoassay/multiplex bead array, LEGENDplex™ Human Growth Factor Panel (13-plex) (Cat. 740180, BioLegend, San Diego, CA, USA), following manufacturer’s instructions. FACS Aria III flow cytometer (BD, Franklin Lakes, NJ, USA) and the LEGENDplex™ Data Analysis Software v 7.0 (BioLegend, San Diego, CA, USA) were used to determine serum levels of both growth factors. The lower limits of detection of factors assayed in the study were EGF (2.6 pg/mL) and VEGF (11.5 pg/mL). The intra-assay coefficients were ≤8.3%, and the inter-assay coefficients of variation were <12.9%.

### 4.3. Quantification of BDNF in Serum

Serum BDNF concentrations were measured using a colorimetric enzyme immunoassay (ChemiKine Brain Derived Neurotrophic Factor Sandwich ELISA Kit, Cat. CYT 306, Sigma-Aldrich, Burlington, MA, USA). Intra- and inter-assay variation coefficients were <10%, respectively. All assays were performed in a double-blind fashion, without knowledge of the clinical status of the sample analyzed.

### 4.4. Statistical Analysis

Clinical and gestational features of participants are described as mean ± SD, while gestational-week periods and sociodemographic characteristics are reported as percentages (%). For continuous variables, Tukey-adjusted pairwise comparisons were applied. Categorical variables were compared using Pearson’s chi-square test.

Pearson’s or Spearman’s rank correlations were computed to assess associations among biochemical parameters, psychometric scores, and clinical variables collected from the tested groups.

An unpaired t-test with Welch’s correction was used to detect differences in the mean serum levels of vascular and neurotrophic growth factors between the anxiety-related symptomatic pregnant population and the healthy control group.

ANOVA with the post hoc Tukey test was used to assess differences in blood levels of vascular and neurotrophic growth factors, clinical variables, and psychometric scores among the study groups. ANOVA with Games–Howell adjustment was applied when variances were not equal, after Levene’s test.

The Friedman test and the chi-square test were used to assess differences in clinical and demographic parameters among pregnant women.

General linear models were constructed using multiple linear regression to analyze whether scores of anxiety (HARS) and depression (HDRS) were predictors of serum BDNF, VEGF, or EGF concentrations. To reduce the influence of heteroscedasticity and non-normal residuals, standard errors were estimated using robust procedures, and confidence intervals were estimated using bias-corrected and accelerated bootstrap with 10,000 resamples. Following the results obtained, an approximate sensitivity analysis was conducted using G*Power 3.1.9.7 version [63], under standard linear regression assumptions to determine the minimum detectable effect size at a chosen power level.

For all statistical analyses, the *p*-value was set at <0.05. All analyses were performed using GraphPad Prism 7 (GraphPad Software Inc., Boston, MA, USA) and SPSS software v.27 (Armonk, NY, USA: IBM CORP) OR JAMOVI 2.7.31.0.

## 5. Conclusions

The present study provides evidence that antenatal anxiety and depressive symptoms are differentially associated with some of the multiple circulating growth factors in late pregnancy. Particularly, the opposite relationship between BDNF and affective vs. anxiety symptoms deserves further consideration, suggesting that anxiety and depression, despite their frequent comorbidity during pregnancy, may engage distinct and potentially opposing components of neurotrophic and vascular regulation. Although the modest proportion of explained biological variance identified precludes causal or biomarker-level interpretations, the current findings broaden the biological framework through which antenatal mental health may be investigated, implying that BDNF, EGF, and VEGF should be regarded as part of interconnected neurotrophic, vascular, and placental adaptive pathways rather than as separate peripheral indicators of psychopathology. Longitudinal studies that incorporate repeated psychometric assessments, maternal circulating factors, placental measures, and obstetric and neonatal outcomes are required to determine the temporal and clinical significance of these associations. Such research may ultimately enable a more precise characterization of the biological heterogeneity underlying anxiety and depression during pregnancy.

## Figures and Tables

**Figure 1:**
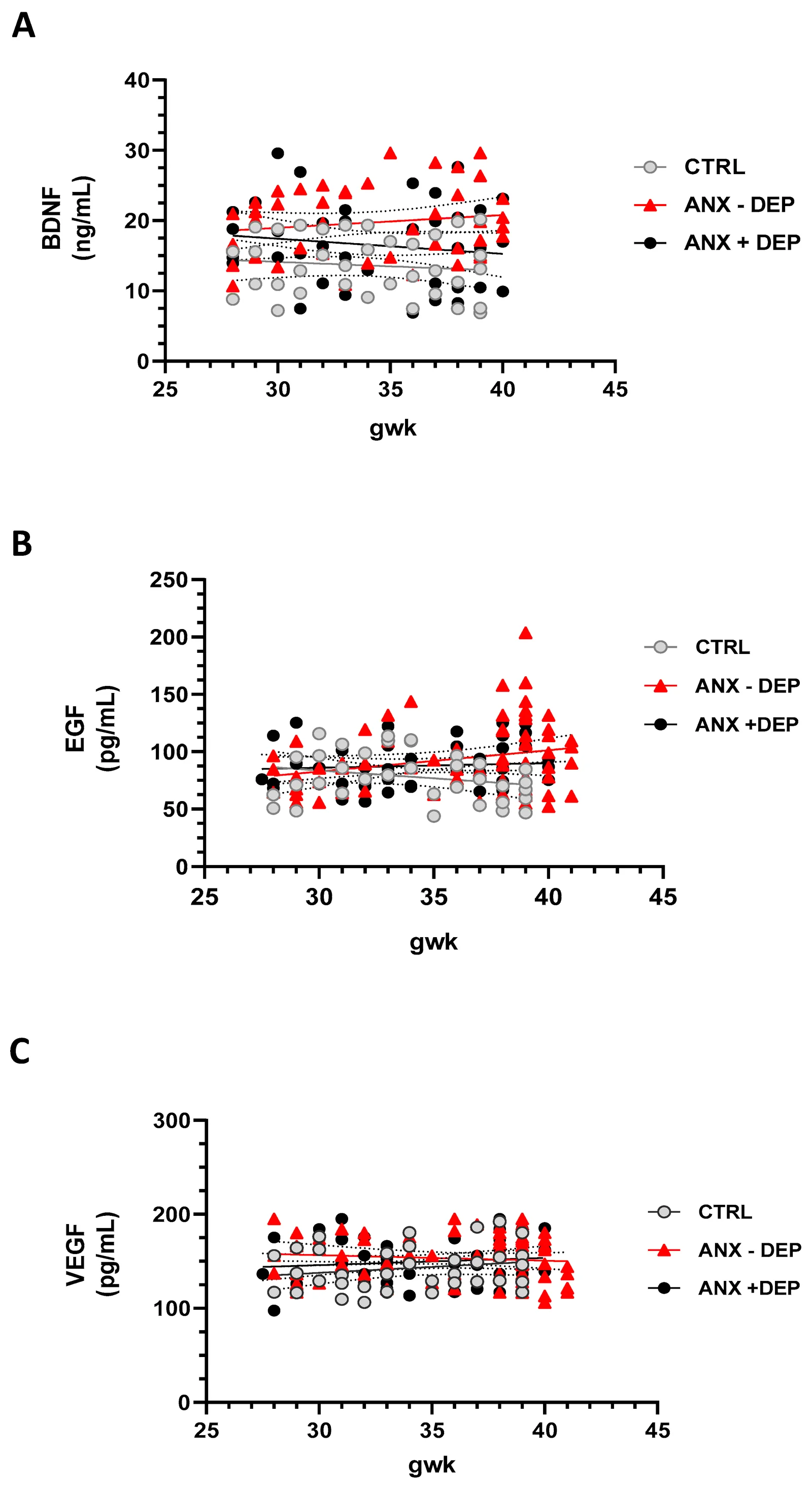
Scatter plots of serum levels for the following factors measured across the third trimester of pregnancy in the symptomatic (ANX − DEP, patients exhibiting severe anxiety; ANX + DEP, patients exhibiting severe anxiety plus comorbid depression) and control (CTRL) groups (28–40 weeks of gestation): (**A**) brain-derived neurotrophic factor (BDNF); (**B**) epidermal growth factor (EGF); and (**C**) vascular endothelial growth factor (VEGF). Dots in each scatter plot represent the individual serum levels of these factors quantified at each gestational week. Regression analyses are as follows. (**A**) *BDNF*: CTRL: R^2^ = 0.0001; F = 0.0005; *p* = 0.98; ANX − DEP: R^2^ = 0.019; F = 1.28; *p* = 0.26; ANX + DEP: R^2^ = 0.013; F = 0.58 *p* = 0.45. (**B**) *EGF*: CTRL: R^2^ = 0.05; F = 1.91; *p* = 0.17; ANX − DEP: R^2^ = 0.13; F = 4.2; *p* = 0.04; ANX + DEP: R^2^ = 0.03; F = 0.26; *p* = 0.61. (**C**) *VEGF*: CTRL: R^2^ = 0.01; F = 0.37; *p* = 0.54; ANX − DEP: R^2^ = 0.02; F = 1.008; *p* = 0.32; ANX + DEP: R^2^ = 0.02; F = 0.82; *p* = 0.37. Abbreviations: gwk, gestation weeks; ANX − DEP, patients exhibiting severe anxiety; ANX + DEP, patients exhibiting severe anxiety plus comorbid depression; CTRL, control group.

**Figure 2:**
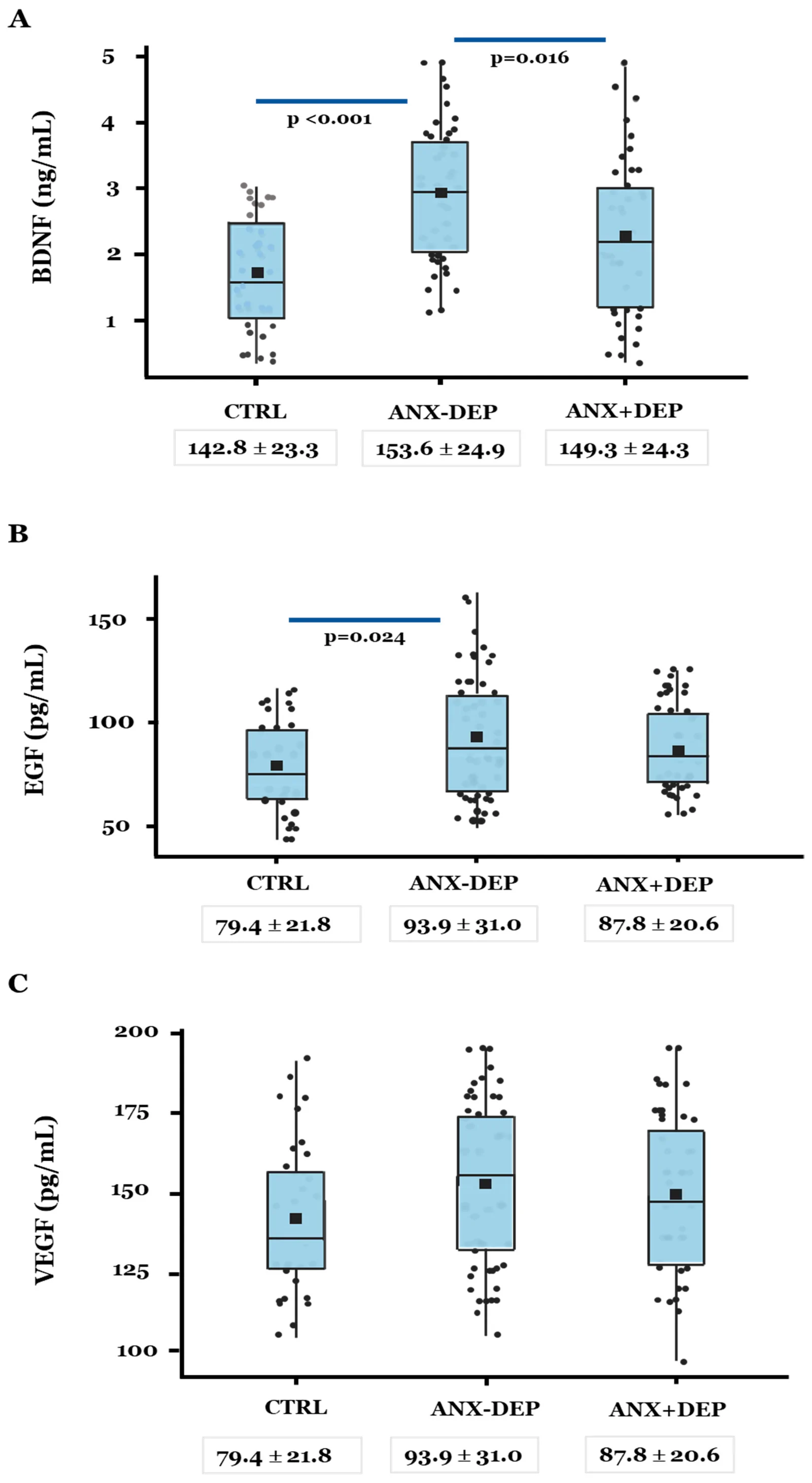
Box plots of the distribution of peripheral serum levels of (**A**) brain-derived neurotrophic factor, (**B**) epidermal growth factor, and (**C**) vascular endothelial growth factor among the study groups during the third trimester of pregnancy. Boxes indicate the interquartile range (IQR; 25th–75th percentiles), the horizontal line within each box represents the median, and whiskers indicate the dispersion of the data beyond the IQR. Black squares represent the group means. Dots illustrate individual values. Horizontal blue bars indicate statistically significant between-group comparisons, with the corresponding *p*-values shown above each comparison. Abbreviations: ANX − DEP, patients exhibiting severe anxiety; ANX + DEP, patients exhibiting severe anxiety plus comorbid depression; CTRL, control group. Inset values displayed below the graph represent the mean ± standard deviation for each clinical category.

**Table 1:** Clinical and gestational characteristics of participants by study group.

*Clinical Characteristics*			
Age, years	28.2 (5.8)	23.8 (5.6) ***	25.0 (5.9) *
Gestational age, weeks	33.9 (3.6)	35.7 (4.1)	34.3 (3.9)
Weight, kg	67.5 (9.8)	67.0 (8.9)	67.2 (11.6)
BMI, kg/m^2^	27.9 (3.8)	27.9 (3.6)	27.4 (3.9)
HARS score	3.1 (0.6)	26.0 (6.3) ***	26.5 (5.7) ***
HDRS score	5.1 (0.9)	5.9 (1.0)	30.1 (7.8) ***
CTRL = control; ANX − DEP = anxiety, no depression; ANX + DEP = anxiety and depression; BMI = body mass index; HARS = Hamilton Anxiety Rating Scale; HDRS = Hamilton Depression Rating Scale. Data are means (standard deviations); *p*-values = * *p* < 0.05, *** *p* < 0.001—represent Tukey-adjusted pairwise comparisons using CTRL as reference			
***Gestational-age*** ***three-week period***			
27–29	8 (22.8)	11 (16.7)	6 (12.8)
30–32	14 (40.0)	12 (18.2)	9 (19.1)
33–35	10 (28.6)	9 (13.6)	12 (25.5)
36–38	3 (8.6)	18 (27.3)	16 (34.1)
39–41	0 (0.0)	16 (24.2)	4 (8.5)

Total N = 148. Data are presented as the nnumber of individuals (percentage) in each group. Distributions of gestational-age three-week periods were compared using Pearson’s chi-square test: χ^2^ (4) = 9.72 *p* = 0.020.

**Table 2:** Sociodemographic characteristics of participants by study group.

Characteristic	CTRL (n = 35)	ANX − DEP (n = 66)	ANX + DEP (n = 47)
***Marital status***			
Never married	6 (17.1)	11 (16.7)	8 (17.0)
Married	5 (14.3)	9 (13.6)	12 (25.5)
Divorced	11 (31.4)	18 (27.3)	10 (21.3)
Cohabiting	13 (37.2)	28 (42.4)	17 (36.2)
***Education level***			
Elementary school	3 (8.6)	6 (9.1)	3 (6.4)
Middle school	12 (34.3)	18 (27.3)	19 (40.4)
High school	10 (28.6)	23 (34.8)	15 (32.0)
Bachelor’s degree	7 (20.0)	10 (15.1)	5 (10.6)
Postgraduate	1 (2.8)	4 (6.1)	3 (6.4)
Technician degree	2 (5.7)	5 (7.6)	2 (4.2)
***Employment status***			
Employed	10 (28.6)	8 (12.1)	12 (25.6)
Unemployed	3 (8.6)	6 (9.0)	2 (4.2)
Home labor	13 (37.1)	32 (48.5)	18 (38.3)
Commerce	6 (17.1)	11 (16.7)	13 (27.7)
Profession	3 (8.6)	7 (10.7)	2 (4.2)
Other	0 (0.0)	2 (3.0)	0 (0.0)

Note. Total N = 148. Data are n (%). CTRL = control; ANX − DEP = anxiety without depression; ANX + DEP = anxiety plus comorbid depression.

## Data Availability

All datasets used or analyzed during the current study are available from the corresponding author upon reasonable request.
